# Amygdala-putamen connectivity links gratitude to greater well-being

**DOI:** 10.1080/19585969.2026.2653597

**Published:** 2026-04-21

**Authors:** Guanmin Liu, Ying Yang, Fei Wang, Feng Kong, Kaiping Peng, Jie Sui

**Affiliations:** a Institute of Applied Psychology, Tianjin University, Tianjin, China; b Academy of Medical Engineering and Translational Medicine, Tianjin University, Tianjin, China; c School of Education, Tianjin University, Tianjin, China; d Department of Psychological and Cognitive Sciences , Tsinghua University, Beijing, China; e School of Psychology, Shaanxi Normal University, Xi’an, China; f School of Psychology, University of Aberdeen, Aberdeen, UK

**Keywords:** gratitude, resting-state functional connectivity, affective well-being, nucleus accumbens, amygdala, putamen

## Abstract

**Introduction:**

Gratitude plays a crucial role in promoting affective well-being, yet the neural mechanisms underlying their relationship remains unclear. Given the central role of the nucleus accumbens (NAcc) and amygdala in emotion and reward processing, this study investigated whether resting-state functional connectivity (RSFC) of these subcortical regions correlates with trait gratitude and mediates its association with affective well-being.

**Methods:**

Resting-state fMRI data were collected from 363 young adults. Seed-based connectivity analyses identified NAcc and amygdala connectivity patterns associated with trait gratitude. Mediation analyses tested whether these patterns explained the association between gratitude and affective well-being.

**Results:**

Trait gratitude was linked to stronger connectivity between the left NAcc and dorsomedial prefrontal cortex and left posterior superior temporal sulcus; between the right NAcc and left dorsolateral prefrontal cortex (DLPFC), right inferior temporal gyrus and bilateral fusiform; and between the right amygdala and right DLPFC, superior temporal gyrus, cerebellum, and putamen. Critically, the right amygdala-putamen connectivity mediated the relationship between gratitude and positive affective well-being.

**Conclusions:**

The right amygdala-putamen RSFC links gratitude to greater affective well-being. This finding identifies a specific subcortical pathway through which grateful dispositions translate into emotional benefit and suggests a potential target for interventions aimed at improving mental health.

## Introduction

Gratitude contributes to affective well-being (Emmons & McCullough, 2003; Watkins et al. 2003; Wood et al. 2010), characterised by more positive affect and less negative affect (Diener et al. 1999; Watson et al. 1988). While converging evidence has supported this relationship (Datu et al. 2022; Sheldon & Lyubomirsky, 2006; Wood et al. 2010), the neural mechanisms underlying their relationship remains largely unknown. Elucidating the neural mechanisms is critical not only for advancing our knowledge of how gratitude supports affective well-being, but also for informing the development of more effective interventions to enhance mental health.

Prior neuroimaging studies have frequently identified the medial prefrontal cortex (MPFC) as important for processing grateful experiences (Fox et al. 2015; Karns et al. 2017; Kini et al. 2016; Liu et al. 2020; Yu et al. 2018), while whole-brain analyses have also implicated additional cortical and subcortical regions. However, this research has focused predominately on transient grateful states evoked by experimental tasks rather than stable grateful dispositions, i.e., trait gratitude, that characterise individuals across contexts and time. Task-based paradigms effectively isolate specific processes but are less suited to capturing the **trait-level individual differences** that determine whether some people consistently experience more gratitude than others. Trait gratitude reflects a stable appraisal style - grateful individuals more readily notice and appreciate positive aspects of their experience. Understanding its neural basis requires methods capable of revealing stable patterns of brain organisation rather than fleeting task-evoked responses.

Resting-state functional connectivity (RSFC) offers such a method. By measuring spontaneous coherence in neural activity across brain regions during undirected rest, it reveals the brain’s intrinsic functional architecture, patterns of coupling that reflect stable individual differences in cognition and affect (Fulwiler et al. 2012; Nostro et al. 2018). While task-based studies have primarily focused on cortical regions, particularly medial prefrontal cortex (MPFC), emerging evidence indicates that subcortical structures exert stronger influence on cortical activity during rest than vice versa (Moazeni et al. 2024). The current study thus focused on two key subcortical regions—the nucleus accumbens (NAcc) and the amygdala—whose roles in emotion and reward make them plausible candidates for supporting grateful dispositions (Davis & Whalen, 2001; Salgado & Kaplitt, 2015).

The nucleus accumbens (NAcc), the principal component of ventral striatum, encodes reward value and motivational salience. Theoretical accounts propose that gratitude enhances affect well-being by intensifying reward signals associated with positive experiences (Watkins et al. 2022). At the neural level, this amplification likely involves enhanced communication between the NAcc and the MPFC which integrates this information into conscious grateful experience (Yu et al. 2018). Grateful individuals might have stronger intrinsic coupling between these regions, facilitating more frequent grateful responses to daily benefits. Consistent with this, task-based studies have linked the NAcc-MPFC connectivity to affective well-being (Heller et al. 2013a; Heller et al. 2009). However, whether NAcc RSFC links to trait gratitude and mediates its relationship with affective well-being remains untested.

The amygdala is a rapid detector of cues affecting well-being (Whalen et al. 1998). It is more sensitive to negative than positive stimuli (Hariri et al. 2000; Monk et al. 2008; Phelps et al. 2001), and this hyperactivity in response to negative stimuli plays a role in depression (Siegle et al. 2007; Zotev et al. 2016). Yet the amygdala does not operate in isolation - the MPFC regulates its responses to aversive stimuli (Motzkin et al. 2015), with stronger amygdala-MPFC connectivity predicting lower anxiety in depressed patients (Mehta et al. 2018). Trait gratitude inversely predicts negative affect and depression (Cregg & Cheavens, 2021; Datu et al. 2022; Wood, Maltby, Gillett, et al. 2008), and grateful individuals showed larger amygdala volume and attenuated amygdala responses to threats (Hazlett et al. 2021; Tani et al. 2022). This suggests that trait gratitude might involve enhanced connectivity between amygdala and regulatory regions, enabling more effective modulation of negative emotional responses.

To date, the relationship between trait gratitude and RSFC of these subcortical regions remains unexplored. One study (Kyeong et al. 2017) examined whether a brief gratitude meditation altered the NAcc- or amygdala-based RSFC but found no effects, unsurprising given that a single five-minute meditation session would be unlikely to reshape stable brain connectivity. More fundamentally, that study examined changes from an intervention rather than trait level individual differences in grateful disposition. The intrinsic neural architecture of trait gratitude, the stable patterns of brain connectivity that distinguish more from less grateful individuals, has yet to be characterised.

### The current study

We addressed these gaps by examining whether resting-state connectivity of NAcc and amygdala is associated with individual differences in trait gratitude, and mediates the relationship between trait gratitude and affective well-being. Based on previous findings on (1) the critical role of MPFC in gratitude (Fox et al. 2015; Karns et al. 2017; Kini et al. 2016; Kong et al. 2020; Liu et al. 2020; Yu et al. 2018) and functional connectivity between MPFC and NAcc/amygdala in affective well-being (Heller et al. 2013a; Heller et al. 2009; Mehta et al. 2018), as well as (2) the engagement of regions associated with mentalizing (e.g. the dorsomedial prefrontal cortex [DMPFC], the posterior superior cingulate cortex [pSTS]) and emotion regulation (e.g. the dorsolateral prefrontal cortex [DLPFC]) in gratitude (Fox et al. 2015; Kini et al. 2016; Kong et al. 2020; Liu et al. 2020; Liu et al. 2018) and affective well-being (Heller et al. 2013b; Kong, Hu, Xue, et al. 2015), we tested two hypotheses:

1. Trait gratitude would correlate with greater connectivity between the bilateral accumbens/amygdala and the cortical regions mentioned above.
2. These connectivity patterns would further account for (mediate) the relationship between trait gratitude and greater affective well-being.

Rather than restricting our search to predicted regions, we employed seed-based whole-brain analyses to identify, without spatial constraint, connectivity correlates of trait gratitude. This approach integrates hypothesis-driven seed selection with whole-brain connectivity mapping, allowing unexpected patterns to emerge while remaining grounded in theoretical motivation for examining particular subcortical structures.

## Methods

### Participants

A total of 363 right-handed university students (189 females; mean age ± SD = 22.52 ± 2.77, aged from 18 to 34) without severe neurological or psychiatric conditions participated in the study. Sample size was not pre-determined, but a post-hoc power analysis using G* Power 3.1 (Faul et al. 2007) indicated that this sample size was sufficient to detect an effect of *r* = 0.19 (corresponding to the weakest association among the variables included in the mediation model, i.e., the association between the right amygdala-putamen RSFC and affective well-being), with 80% power at α = 0.05. All the participants were recruited through online advertisement. All participants gave informed consent and the protocols were approved by the Institutional Review Board of the School of Medicine at Tsinghua University (Protocol number: 20140066).

### Measures

*Trait gratitude* was measured with the most widely used instrument, the Gratitude Questionnaire-6 (GQ-6) (McCullough et al. 2002). The scale consists of 6 items, such as “I have so much in life to be thankful for”. Participants rated the items using a 7-point Likert scale from 1= strongly disagree to 7 = strongly agree. The scale was reliable (Cronbach’s α = 0.78). *Affective well-being* was measured with the Positive and Negative Affect Schedule (PANAS) (Diener et al. 1999; Watson et al. 1988). The scale consists of two subscales: positive affect and negative affect. Each subscale consists of 10 items of emotional adjectives. Participants rated the extent to which they have felt each emotion recently using a 5-point Likert scale from 1= very slightly or not at all to 5 = extremely. Affective well-being was calculated by summing up the scores of positive items and reversed scores of negative items (Kong, Hu, Wang, et al. 2015). Both dimensions were reliable (positive: α = 0.87; negative: α = 0.86). Note that four out of the 363 participants did not finish the scale, and they were excluded from further correlation and mediation analyses where affective well-being was involved.

### Image acquisition

Imaging data were acquired on a 3.0 T Philips Achieva 3.0 T TX system with a SENSE 8-channel head coil at the Centre of Biomedical Engineering, Tsinghua University. Functional images were acquired with a T2-weighted echo-planar imaging sequence (TR = 2,300 ms, TE = 30 ms, flip angle = 90°, 37 ascending slices, FOV = 256 × 256 cm, acquisition matrix = 96 × 96 × 37, voxel size = 2.5 mm × 2.5 mm × 3.45 mm). Participants were instructed to keep their eyes open during the functional scan and avoid thinking about anything in particular or falling asleep. The total duration of the resting-state scan was 508.3 s. T1-weighted structural images were acquired with TR = 8.2 ms, TE = 3.8 ms and flip angle = 8°. The SENSE factor was 2/1.5 for AP/RL, and the acquisition matrix was 256 mm × 256 mm. A total of 160 contiguous sagittal slices were acquired with a voxel size of 0.938 mm × 0.938 mm × 1 mm.

### Image pre-processing

The resting-state images were preprocessed with Data Processing Assistant for Resting-State fMRI (DPARSF), version 5.0 (Yan & Zang, 2010). The first ten volumes were discarded. The rest volumes were slice-timing and head motion corrected, and framewise displacement (FD) was computed (Jenkinson et al. 2002). Head motion was controlled by including motion parameters as nuisance regressors and by accounting for mean FD at the group level (no participants were excluded solely based on motion parameters). The T1-weighted images were then segmented using the New Segmentation module. The resulting c1 images were submitted to the Diffeomorphic Anatomical Registration Through Exponentiated Lie Algebra (DARTEL) procedure (Ashburner, 2007) to create a sample-specific, normalised-to-the-MNI-space grey matter template. Nuisance regression was then performed on the functional images, the covariates of which included Friston et al. (1996)’s 24 head motion parameters, 5 principal components of signals of white matter/CSF (Behzadi et al. 2007), and a linear term (i.e., detrending). The global signal was not regressed out to avoid the introduction of artificial anticorrelations (Murphy et al. 2009). The resulting images were normalised to the MNI space using the DARTEL procedure and smoothed with a Gaussian kernel of full width at half maximum (FWHM) equal to 4 mm, during which the voxels were resized to 3 mm × 3 mm × 3 mm. Lastly, the functional images were bandpass filtered with 0.01–0.10 Hz.

### Imaging data analysis

#### Seed region definition

The seed regions were defined using the Wake Forest University Pickatlas Toolbox (Maldjian et al. 2003) by the labels of bilateral NAcc in the 71-segmentation version of the Individual Brain Atlases Using Statistical Parametric Mapping (IBASPM), and by the labels of bilateral amygdala in the 116-segmentation version of IBASPM. For each seed region, the mean BOLD time series was extracted by averaging signals across all voxels within the anatomically defined ROI. Voxel-wise Pearson correlations were then computed between the seed time series and every voxel in the brain to construct individual-level RSFC maps. The resulting correlation coefficients were Fisher-z transformed to improve normality. Second-level analyses were subsequently performed on the correlation maps using SPM 12. A grey matter mask was created by binarizing SPM’s prior probability grey matter map at the threshold of 0.2 and was applied to second-level analyses.

#### Seed-based whole-brain connectivity analyses

To examine whether trait gratitude correlated with any RSFC of bilateral NAcc and amygdala, regression models were performed on each of the seed-based Z-maps, with trait gratitude as predictor, and age, gender, and FD as covariates. Whole-brain analyses were conducted with the cluster-extent threshold of *p_FWE_* < 0.05 and the voxel-height threshold of *p_uncorr_* < 0.001. Calculation using Monte Carlo and 3dClustSim in AFNI 18 (https://afni.nimh.nih.gov/) yielded a minimum cluster size of 32 voxels. We further extracted the eigenvalues of the significant clusters for further correlation and mediation analyses.

#### Mediation analysis

To examine whether NAcc- or amygdala-based RSFC correlates of trait gratitude (couplings with any regions identified in the above-mentioned regression models) would mediate the relationship between trait gratitude and affective well-being, the following analyses were conducted. First, we conducted correlational analyses between the NAcc-/amygdala-based RSFC correlates of trait gratitude and affective well-being. Second, when there was evidence for significant correlation(s) between affective well-being and certain identified RSFC, we tested the hypothesised mediation model using Preacher and Hayes’s (2008) PROCESS package with bias-corrected bootstrapping. Indirect effects were reported with 95% confidence intervals. Bonferroni correction was applied prior to mediation testing to control for multiple comparisons across candidate RSFC mediators.

If any RSFC was identified to mediate the relationship between trait gratitude and affective well-being, the same processes with dependent variable as positive or negative affect would be further conducted to explore whether it is the relationship of this RSFC with positive affect or with negative affect that drives the results for affective well-being.

## Results

### Self-report measures

Correlational analyses showed that trait gratitude correlated with affective well-being, *r* = 0.24, *p* < 0.001. Specifically, the correlation between gratitude and positive affect was *r* = 0.24, *p* < 0.001; while that between gratitude and negative affect was *r* = -0.13, *p* = 0.013.

### Neuroimaging measures

#### Seed-based whole-brain connectivity analyses

**Figure 1.**
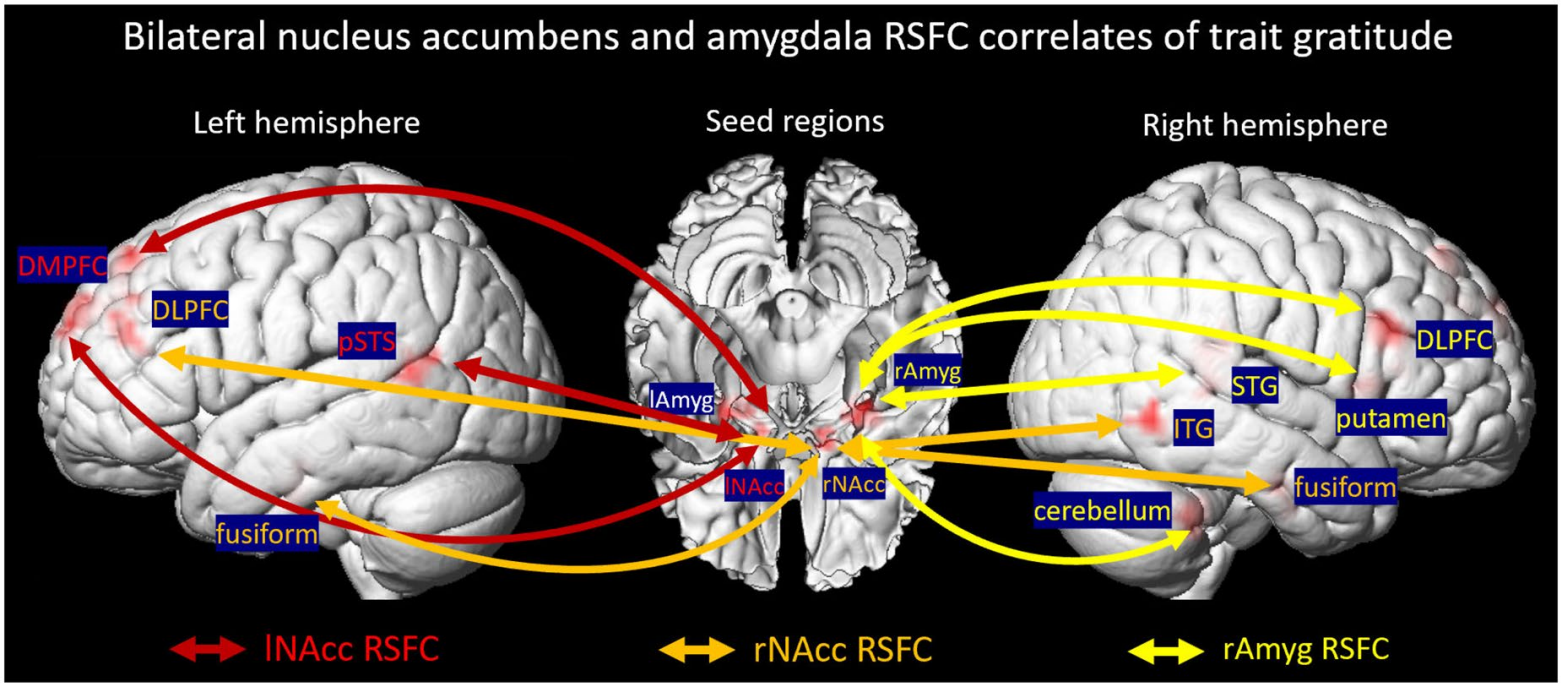
Trait gratitude was positively correlated with the left nucleus accumbens (lNAcc) resting-state functional connectivity (RSFC) with the dorsomedial prefrontal cortex (DMPFC) and the left posterior superior temporal sulcus (pSTS) (in red color), the right nucleus accumbens (rNAcc) RSFC with the left dorsolateral prefrontal cortex (DLPFC), the right inferior temporal gyrus (ITG) and bilateral fusiform (in orange color), as well as the right amygdala (rAmyg) RSFC with the right DLPFC, superior temporal gyrus (STG), cerebellum and putamen (in yellow color). On the other hand, trait gratitude showed no correlation with any left amygdala (lAmyg) RSFC.

The results are presented in Table 1 and Figure 1. The results demonstrated that trait gratitude was positively associated with the left NAcc RSFC with the DMPFC (*r*s = 0.23 for both clusters) and the left pSTS (*r* = 0.20), the right NAcc RSFC with the left DLPFC (lDPFC; *r* = 0.23), the right inferior/middle temporal gyrus (*r* = 0.18), and bilateral fusiform extending to parahippocampal gyrus (left: *r* = 0.23; right: *r* = 0.21), as well as the right amygdala RSFC with the right DLPFC (*r* = 0.22), superior/middle temporal gyrus (*r* = 0.22), cerebellum (*r* = 0.24) and putamen extending to inferior frontal gyrus and insula (*r* = 0.20). Together, these findings indicate that trait gratitude is associated with widespread enhanced connectivity between subcortical reward and salience regions and cortical regions implicated in social cognition and emotion regulation.

**Table 1.** Bilateral accumbens- and amygdala-based resting-state functional connectivity (RSFC) correlates of trait gratitude in whole-brain analyses. (*p_uncorr_* < .001 at voxel level, cluster-level *p_FWE_* < .05).

Region	Cluster Size (K)	Hemisphere	MNI coordinates			*Z*
			x	y	z	
**Left NAcc as seed region**						
DMPFC (SFG), BA10/9	50	Left	−12	57	30	4.64
DMPFC (SFG), BA8/9/6	100	Left/Right	−6	39	48	4.10
pSTS (MTG), BA22/39/21	50	Left	−63	−54	9	3.97
**Right NAcc as seed region**						
DLPFC (MFG), BA46/10	33	Left	−42	48	30	4.82
Fusiform/PHG/ITG, BA20	43	Right	42	−15	−27	4.04
Fusiform/PHG/hippocampus, BA35/20	46	Left	−24	−15	−33	3.96
ITG/MTG, BA37/21	34	Right	66	−60	−6	3.95
**Left amygdala as seed region**						
No suprathreshold clusters						
**Right amygdala as seed region**						
DLPFC (IFG/MFG), BA46	77	Right	45	24	24	5.10
Cerebellum	41	Right	33	−42	−36	4.02
Putamen/IFG/insula, BA13	39	Right	27	18	6	3.84
STG/MTG, BA41/40	51	Right	54	−33	21	3.81

DMPFC, dorsomedial prefrontal cortex; SFG, superior frontal gyrus; pSTS, posterior superior temporal sulcus; MTG, middle temporal gyrus; MFG, middle frontal gyrus; PHG, parahippocampal gyrus; DLPFC, dorsolateral prefrontal cortex; IFG, inferior fusiform gyrus.

All RSFCs in the table positively correlated with trait gratitude.

#### Mediation analyses

We next tested whether any of these connectivity patterns accounted for the relationship between gratitude and affective well-being. Several patterns correlated with affective well-being: the right amygdala-DLPFC (*r* = 0.13, *p* = 0.018), the right amygdala-putamen (*r* = 0.19, *p* < 0.001), the left NAcc-pSTS (*r* = 0.14, *p* = 0.007), the right NAcc-lDLPFC (*r* = 0.11, *p* = 0.042), and the right NAcc-inferior temporal gyrus (*r* = 0.13, *p* = 0.014). To control for multiple comparisons, we applied a Bonferroni correction, and only the correlation between affective well-being and the right amygdala-putamen RSFC remained significant (Bonferroni-corrected *p* = 0.004). Thus, across the 11 candidate pathways, only the right amygdala–putamen connectivity emerged as the most robust connectivity correlate of affective well-being.

**Figure 2.**
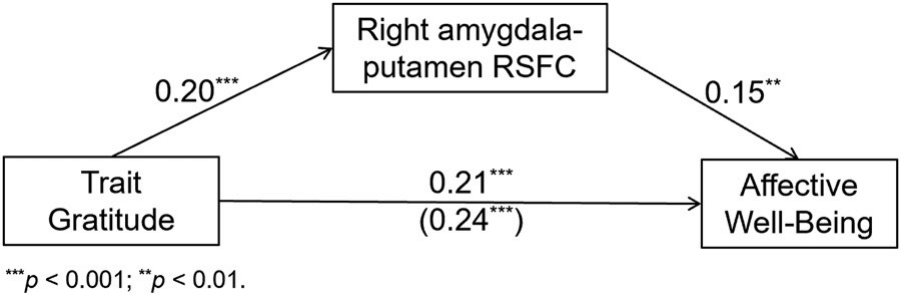
The right amygdala-putamen resting-state functional connectivity (RSFC) partially explained the relationship between trait gratitude and affective well-being.

Formal mediation analysis confirmed that the right amygdala-putamen RSFC partially account for the link between trait gratitude and affective well-being. Figure 2 showed that the indirect effect of trait gratitude on affective well-being *via* the right amygdala-putamen was significant (95% CIs: [0.02, 0.13]). These results indicate that *greater right amygdala-putamen RSFC* partially explained the relationship between *trait gratitude* and *affective well-being*.

Further correlation analyses showed that the right amygdala-putamen RSFC was significantly correlated with positive affect (*r* = 0.20, *p* < 0.001), but not negative affect (*r* = -0.09, *p* = 0.082)^1^. Mediation analysis showed that the indirect effect of trait gratitude on positive affect *via* the right amygdala-putamen was significant (95% CIs: [0.02, 0.08]). These results suggest that the relationship between trait gratitude, amygdala-putamen RSFC and affective well-being was primarily driven by that between trait gratitude, amygdala-putamen RSFC and *positive affect*.

## Discussion

This study sought to identify the intrinsic neural architecture of trait gratitude and test whether specific connectivity patterns mediate the relationship between trait gratitude and affective well-being. Three principal findings emerged. First, individual differences in trait gratitude were associated with distinct connectivity patterns: between the left NAcc and the DMPFC and left pSTS; between the right NAcc and the left DLPFC, right inferior/middle temporal gyrus and bilateral fusiform/parahippocampal gyrus; and between the right amygdala and the right DLPFC, superior/middle temporal gyrus, cerebellum, and putamen/inferior prefrontal gyrus/insula. Second, among these patterns, connectivity between right amygdala and putamen most robustly predicted affective well-being. Third, this amygdala-putamen pathway statistically mediated the relationship between trait gratitude and affective well-being, accounting particularly for enhanced positive rather than diminished negative affect. Together, these findings illuminate how grateful dispositions are instantiated in stable brain organisation, suggesting a specific subcortical circuit through which gratitude confers emotional benefit.

### Trait gratitude and NAcc connectivity

Enhanced connectivity between the left NAcc and DMPFC in more grateful individuals is consistent with theoretical accounts that gratitude involves integrating reward signals with social understanding (Fox et al. 2015). DMPFC is engaged in mentalizing (Gallagher & Frith, 2003; Sui et al. 2024; Van Overwalle, 2009; Van Overwalle & Baetens, 2009), inferring others’ mental states and intentions. Stronger coupling between this region and NAcc may be associated with a greater tendency to experience gratitude in response to perceived benevolence from others. This interpretation gains support from our finding that trait gratitude was also positively correlated with NAcc RSFC with pSTS, fusiform/parahippocampal gyrus, and inferior/middle temporal gyrus – regions consistently implicated in social cognition.

As a social emotion, gratitude engages regions associated with social cognition and especially mentalizing, such as the temporal cortex (Ding et al. 2014). The temporal cortex of pSTS, fusiform or inferior temporal gyrus is involved in gratitude (Fox et al. 2015; Liu et al. 2020; Liu et al. 2018; Zahn et al. 2014; Zahn et al. 2007). These regions are all involved in social cognition. Among them, pSTS is a critical region in mentalizing, mirror system, and social salience (Gallagher & Frith, 2003; Liang et al. 2022; Sui et al. 2015; Sui & Gu, 2017; Sui et al. 2013; Van Overwalle, 2009; Yankouskaya et al. 2022; Zhang et al. 2026; Zhang et al. 2023). While the fusiform is overwhelmingly associated with face processing (Kanwisher et al. 1997), some studies showed that it was also engaged in social cognition such as decoding communicative intentions (Wang et al. 2006) and self-other discrimination (Shi et al. 2021; Sui et al. 2012). Likewise, the inferior temporal gyrus was also involved in social cognition such as attribution of intentions to others (Brunet et al. 2000). A previous fMRI study showed that generous intention not only activated regions associated with mentalizing, but also reward-related regions (Liu et al. 2020). These findings suggest that the relationship between trait gratitude and the NAcc RSFC with mentalizing-related regions might be explained by grateful individuals’ stronger reward response to others’ generous intentions.

We also found a positive correlation between trait gratitude and the NAcc-DLPFC RSFC. Previous research has shown that the functional connectivity between NAcc and DLPFC is associated with the process of generating and sustaining positive emotions through regulation strategies (Heller et al. 2013b; Weng et al. 2013). Given that gratitude helps sustain positive emotions (Sheldon & Lyubomirsky, 2006), the enhanced NAcc-DLPFC RSFC in grateful individuals may reflect a stronger capacity to generate and sustain positive emotions through cognitive strategies such as re-appreciation (Bao & Lyubomirsky, 2014).

### Trait gratitude and amygdala RSFC

The association between trait gratitude and the right amygdala-DLPFC RSFC is particularly noteworthy given the DLPFC’s role in emotion regulation (Etkin et al. 2015; Goldin et al. 2008; Ochsner et al. 2012). According to Etkin et al. (2015), DLPFC and MPFC are engaged in explicit and implicit emotion regulation, respectively. This connectivity pattern may reflect a greater capacity for deliberate regulation of negative emotions in grateful individuals. Conversely, the lack of a significant association between trait gratitude and amygdala-MPFC connectivity may suggest that grateful individuals tend to rely more on explicit rather than implicit emotion regulation strategies.

Furthermore, the greater right amygdala RSFC with the superior/middle temporal gyrus, cerebellum, and putamen in grateful individuals may reflect more sophisticated social emotional processing capacities. While the amygdala plays an important role in emotional learning, the superior temporal gyrus is involved in representing dynamic features of faces (Adolphs, 2002), and the cerebellum contributes to social mirroring and mentalizing processing (Van Overwalle et al. 2014; Van Overwalle et al. 2020). These connectivity patterns may enable grateful individuals to interpret socio-affective cues more effectively and to generate appropriate grateful responses to others’ generous behaviours.

The association between gratitude and the right amygdala-putamen RSFC is somewhat intriguing. The putamen is associated with reward processing, especially reward prediction error (Bartra et al. 2013; O’Doherty et al. 2004), and is engaged in gratitude (Liu et al. 2020). While the amygdala responds more reliably to negative emotions, it also responds to rewards and is part of the reward circuit (Baxter & Murray, 2002; Murray, 2007). More specifically, it plays a role in stimulus-reward association learning, i.e., linking neutral stimuli with reinforcers such as foods (Murray, 2007). This role is especially relevant to the function of trait gratitude, which predicts higher perceived subjective value in a certain benefit (Wood, Maltby, Stewart, et al. 2008). Grateful individuals may be better at appreciating things whose value is usually unnoticed, which can be seen as a process of linking neutral stimuli (things whose value is usually unnoticed) with reinforcers (important subjective value attached to the things *via* appreciation), and thus have greater amygdala-putamen RSFC.

Notably, all observed effects involving the amygdala were specific to the **right hemisphere**, suggesting a lateralised involvement in trait gratitude. Previous research has shown that the right amygdala is more involved in automatic detection of emotionally salient stimuli, whereas the left amygdala is associated with more deliberate emotional evaluation of specific stimuli (Gläscher & Adolphs, 2003; Wright et al. 2001). The right-lateralised connectivity observed here may reflect both an increased reflexive emotional responsiveness that supports spontaneous feelings of appreciation (as indicated by the right amygdala-putamen RSFC) and a greater readiness for deliberate regulation of spontaneous negative emotions (as indicated by the right amygdala-DLPFC RSFC) in individuals high in trait gratitude.

### The mediating effect of amygdala-putamen RSFC between trait gratitude and affective well-being

The right amygdala-putamen RSFC was significantly correlated with affective well-being. More importantly, it partially explained the relationship between trait gratitude and affective well-being. Further analyses revealed that these relationships were primarily driven by the positive affect component of affective well-being, suggesting that the right amygdala-putamen RSFC may play a crucial role in the way trait gratitude fosters greater positive affect. The right amygdala-putamen RSFC may serve as a neural basis for spontaneous emotional sensitivity to aspects of everyday life that are commonly overlooked. The association of the right amygdala-putamen RSFC with positive but not negative affect further supports its role in enhancing positive affect rather than attenuating negative affect. Grateful people may be more prone to spontaneously associate neutral stimuli with reward-related signals *via* the right amygdala-putamen pathway, which may contribute to a higher level of affective well-being, particularly positive affect. Nevertheless, this interpretation remains tentative due to the correlational nature of the study. Future research employing longitudinal or experimental designs is needed to establish the directionality and clarify the psychological functions reflected in the observed RSFC mediation.

### Theoretical and methodological contributions

These findings advance understanding of gratitude’s neural substrates in three ways. First, by examining resting-state rather than task-based connectivity, we revealed the intrinsic functional architecture distinguishing grateful from less grateful individuals, patterns present even in the absence of grateful stimuli. This complements task-based studies by showing that gratitude reflects stable brain organisation, not merely transient responses to specific contexts. Second, by systematically examining subcortical structures (NAcc, amygdala) whose resting connectivity has received scant attention in gratitude research, we identified reward-related and emotion-regulatory circuits that theoretical accounts emphasised but prior empirical work has largely neglected. Third, by demonstrating a formal mediation, we moved beyond correlation to test a specific mechanistic right amygdala–putamen pathway, linking trait gratitude to affective well-being. While correlational data cannot establish causation definitively, mediation analysis tests whether patterns consistent with causal models exist in the data, a necessary if insufficient step towards causal understanding. From an applied perspective, identifying a specific amygdala–putamen pathway may also inform the development of gratitude-based or reward-focused interventions aimed at enhancing positive affect and affective well-being.

### Limitations

Several limitations warrant consideration. First, the resting-state functional connectivity analyses are correlational in nature and cannot establish causal relationships between trait gratitude, neural connectivity, and affective well-being. Second, the stepwise mediation analysis employed may be subject to serial test bias. While this bias does not invalidate the analysis, it increases the likelihood of false positives. To mitigate this, we applied a Bonferroni correction, but future studies would benefit from pre-registered hypotheses to validate the findings. Third, while RSFC is well-suited to capturing trait-like neural patterns, it lacks cognitive process isolation afforded by task-based fMRI and cannot reveal brain responses elicited by gratitude-evoking stimuli. Future studies could combine both resting-state and task-based approaches to achieve a more comprehensive understanding. Fourth, participants were instructed to keep their eyes open during scanning but were not required to fixate on a central cross. Although this approach is widely used in the resting-state literature and standard motion correction as well as nuisance regression were applied to reduce potential noise, future studies would benefit from replication under different eye conditions. Lastly, participants were recruited online, which may introduce selection biases and limit the generalisability of the findings to broader populations. Beyond quantitative neuroimaging approaches, future research may also benefit from integrating qualitative or participatory methods (e.g. online photovoice or community-based approaches) to more directly capture individuals’ lived experiences of gratitude and emotional well-being, thereby complementing neural findings with experiential perspectives (Dari et al. 2023; Dari et al. 2025; Doyumğaç, 2020; Tanhan & Strack, 2020; Waalkes et al. 2025).

## Conclusion

Our results reveal that grateful individuals exhibit distinctive patterns of intrinsic brain connectivity between subcortical structures (NAcc, amygdala) processing reward and salience and cortical regions supporting social cognition and emotion regulation. Among these patterns, connectivity between the right amygdala and putamen predicts affective well-being, and mediates the relationship between trait gratitude and affective well-being. This pathway appears to amplify positive emotional responses rather than suppress negative ones, suggesting that gratitude operates primarily by enhancing appreciation of benefits rather than defending against harms. These findings identify a specific neural circuit through which grateful dispositions translate into emotional benefit and suggest that interventions targeting this circuit, whether through psychological training or neuromodulation, might enhance well-being by cultivating more automatic positive responsiveness to benefits in life.

## Data Availability

Data in this article are available upon request.

## Note

1 The results remained essentially unchanged when we correlated all eleven identified RSFCs with positive or negative affect separately. Among all the eleven identified RSFCs, positive affect correlated only with the right amygdala-superior/middle temporal gyrus (*r* = 0.14, *p* = 0.009), the right amygdala-DLPFC (*r* = 0.13, *p* = 0.013), the right amygdala-putamen (*r* = 0.20, *p* < 0.001), and the left NAcc-pSTS (*r* = 0.15, *p* = 0.005). After Bonferroni correction, only the right amygdala-putamen RSFC remained significant (Bonferroni-corrected *p* = 0.002). Negative affect showed no significant correlations with any RSFCs.
